# Effectiveness of a primary care based multifactorial intervention to improve frailty parameters in the elderly: a randomised clinical trial: rationale and study design

**DOI:** 10.1186/1471-2318-14-125

**Published:** 2014-11-27

**Authors:** Laura Romera, Francesc Orfila, Josep Maria Segura, Anna Ramirez, Mercedes Möller, Maria Lluïsa Fabra, Santiago Lancho, Núria Bastida, Gonçal Foz, Maria Assumpta Fabregat, Núria Martí, Montserrat Cullell, Dolors Martinez, Maria Giné, Anna Bistuer, Patricia Cendrós, Elena Pérez

**Affiliations:** Primary healthcare centre Raval Nord, Institut Català de la Salut, Barcelona, Spain; Universitat Autònoma de Barcelona, 08193 Bellaterra, Spain; Institut Universitari d’Investigació en Atenció Primària Jordi Gol (IDIAP Jordi Gol), Barcelona, Spain; Gerència d’Àmbit d’Atenció Primària Barcelona Ciutat, Institut Català de la Salut, Barcelona, Spain; Department of Medicine, Universitat de Barcelona, Barcelona, Spain; Rehabilitation Unit, Drassanes Health Centre, Institut Català de la Salut, Barcelona, Spain; Department of Physical Activity and Sport Sciences, FPCEE Blanquerna, Universitat Ramon Llull, Barcelona, Spain; Social Training Centre Mil.lenari, Universitat Oberta de Catalunya, Vic, Spain; Family and Social Welfare, Institut Català d’Assistència I Serveis Socials (ICASS), Barcelona, Spain

**Keywords:** Frail elderly, Aged, Randomised controlled trial, Exercise, Disability, Primary health care, Treatment outcome

## Abstract

**Background:**

Frailty is a highly prevalent condition in old age leading to vulnerability and greater risk of adverse health outcomes and disability. Detecting and tackling frailty at an early stage can prevent disability. The purpose of this study is to evaluate the effectiveness of a multifactorial intervention program to modify frailty parameters, muscle strength, and physical and cognitive performance in people aged 65 years or more. It also assesses changes from baseline in falls, hospitalizations, nutritional risk, disability, institutionalization, and home-care.

**Methods/design:**

The current study is a randomised single-blind, parallel-group clinical trial, with a one and a half year follow-up, conducted in eight Primary Health Care Centres located in the city of Barcelona. Inclusion criteria are to be aged 65 years or older with positive frailty screening, timed get-up-and-go test between 10 to 30 seconds, and Cognition Mini-Exam (MEC-35) of Lobo greater than or equal to 18. A total of 352 patients have been equally divided into two groups: intervention and control. Sample size calculated to detect a 0.5 unit difference in the Short Physical Performance Battery (Common SD: 1.42, 20% lost to follow-up). In the intervention group three different actions on frailty dimensions: rehabilitative therapy plus intake of hyperproteic nutritional shakes, memory workshop, and medication review are applied to sets of 16 patients. Participants in both intervention and control groups receive recommendations on nutrition, healthy lifestyles, and home risks.

Evaluations are blinded and conducted at 0, 3, and 18 months. Intention to treat analyses will be performed. Multivariate analysis will be carried out to assess time changes of dependent variables.

**Discussion:**

It is expected that this study will provide evidence of the effectiveness of a multidisciplinary intervention on delaying the progression from frailty to disability in the elderly. It will help improve the individual’s quality of life and also reduce the rates of falls, hospital admissions, and institutionalizations, thus making the health care system more efficient. This preventive intervention can be adapted to diverse settings and be routinely included in Primary Care Centres as a Preventive Health Programme.

**Trial registration:**

ClinicalTrials.gov PRS:NCT01969526. Date of registration: 10/21/2013.

## Background

The concept of frailty has long been associated with advancing age although only recently has it been specifically defined as a medical syndrome[1–4]. Disability, comorbidity, malnutrition, biological changes, cognitive impairment, dependency needs, and demands for social services are directly age-related; age, however, as an isolated criterion is not enough to identify vulnerability[5].

Despite considerable discussion, frailty remains to be systematically defined[6]. There is evidence that it increases proportionally to an accumulation of deficits[7]; acute problems -falls, fractures, and infections-, progressive loss of autonomy, and psychosocial limitations all lead to disability and a higher risk of hospitalization, institutionalization, and death[8, 9].

There is no agreement on a valid evaluation model for both research and clinical approaches[10], and some authors differentiate between physical and cognitive frailty[11]. The standard clinical proposal is that which has been presented by Fried *et al*. who identify someone as having a frail phenotype when three or more of the following components are presented[3]: unintentional weight loss (4.5 kg (=10 lbs) in the past year), self-reported exhaustion (two positive questions of Center for Epidemiologic Studies Depression Scale (CES-D)), weakness, slow walking speed, and low levels of physical activity. Such a definition would be mainly related to physical frailty. For rapid frailty screening of a community-living elderly population these five criteria, however, do not represent a pragmatic approach[10]. Avila-Funes *et al*. proposed a review to slightly modify Fried’s measurements in order to strengthen the predictive validity of the concept[12], with variable results[13, 14]. In addition, Gill *et al*. introduced two tests of physical ability strongly associated with disability development and progression: the Rapid-Gait Test and the Stand-Up Test[15]. Other useful batteries of physical performance, such as the Short Physical Performance Battery (SPPB) from Guralnik, can also be found as predictors of old age disability[16, 17].

Sarcopenia is linked to physical frailty and is a key feature of this condition in older people[18]. In fact, a non-negligible proportion of elderly individuals are moderately affected by this it[19]. Sarcopenia is related to loss of muscle mass and muscle strength, strong predictors of adverse health outcomes[20] and death[21, 22].

Epidemiological studies have linked physical frailty and cognitive impairment: frailty increases the risk of cognitive decline and cognitive impairment increases the risk of frailty, therefore, both dimensions would benefit from being addressed[23, 24].

Identifying interventions to prevent or delay the loss of autonomy is currently a public health priority for the successful management of the ageing[25, 26]. Multidimensional home interventions have revealed some benefits, although conclusions are inconsistent and seem to be dependent on factors such as the provider’s experience, access to monitoring, and duration of the follow-up program[15].

A comprehensive geriatric assessment, followed by a multidimensional intervention on disability risk factors -medical, functional, psychological, and environmental problems- through disease management and health promotion in a low-risk elderly population, succeeds in reducing institutionalization and the risk of falls, delaying disabled functional decline, and improving physical performance. The effects, however, are not statistically significant[27–29].

Strategies involving mass screening in Primary Care to apply preventive approaches based on healthy ageing advice, long-term exercise programs, assistance devices including home telecare kits[30], and environmental modifications can reduce falls[31]. Nevertheless, when considered separately, these methods have no impact on reducing disability[32].

Exercise programs improve strength, aerobic capacity, balance, and function[33, 34], but these benefits depend on long-term adherence, extended training, and exercise-related behaviours acquired in early life. The most promising strategies to increase physical activity in the elderly are those which provide appropriate written advice and generate feelings of fun and satisfaction[35].

Recent surveys have put forward new strategies for the management of sarcopenia to slow down the decline of muscle features: resistance training in combination with adequate protein and energy intake and, additionally, treatment of vitamin D deficiency[36, 37].

Nutritional interventions alone show weak correlation with health improvement in the vulnerable, elderly population. However, dietary advice in association with protein supplementation intake seems to have some effects on sarcopenia, inducing muscle hypertrophy, accelerating weight gain in undernourished older people[38], and reducing fractures[39]. There is a lack of evidence, however, concerning its effects on mortality and hospital admission rates[40].

Findings from cognitive training studies show positive effects. Memory training can aid maintaining long-term improvement in performance[41, 42].

Exercise also leads to enhanced cognitive functioning and psychological well-being in frail, older adults[43]. Aerobic exercise has shown effects on some measures of cognitive function, without consistency for all values[44].

There are few randomized, controlled trials concluding that cognitive interventions, plus complementary physical exercise, can produce significant global improvements in cognitive function, and quality of life, and delay the onset of disability[45].

What about medication use in frail, older adults? The rates of adverse drug events are higher in the elderly population, as many of them have comorbidities, multiple drug prescriptions, and deteriorated physical and cognitive impairment[46]. In the previous decade, deprescribing, based on clinical and ethical criteria, has been defended as an option for managing chronic conditions, avoiding adverse effects, and improving patient outcomes. Polypharmacy has been independently associated with an increase of mortality in the elderly[47], indeed, several multifaceted interventional studies have demonstrated that medication review has a positive effect on reducing mortality, hospital admissions and falls, and enhances quality of life[48].

Such a wide range of interrelated factors gives weight to our proposal to conduct a multifactorial intervention aimed at non-disabled, i.e. frail, elderly individuals. Our objective is to focus on this population whose health status still permits some positive modifications in the inevitable evolution from frailty to dependence so that by preventing home confinement or institutionalization, older people can stay active and live by themselves in the community.

### Study aim

This is a research protocol for a randomized, controlled trial aimed at assessing the effectiveness of a multifactorial intervention program to modify parameters of frailty, muscle strength, and physical and cognitive performance in elderly people living in the community. The intervention includes various professional disciplines and is based on physical activity, diet supplementation, memory workshops, and medication review.

Secondary aims include evaluating changes in rates of falls fractures, hospital admissions, inclusions in home care programs, and institutionalizations.

## Methods/design

### Study design

The study design is a single-blind, parallel-group, pragmatic, randomised, clinical trial with one year and a half follow-up.

Changes from baseline measurements (month 0) in the parameters of frailty, muscle strength, and physical and cognitive performance are compared between the intervention (IG) and control group (CG) at the end of the intervention (month 3). An 18 month follow-up after randomization will be established in order to determine whether intervention effects can be sustained. The 18 month changes in rates of falls, fractures, hospital admissions, inclusions in home care programs, institutionalizations and vital status will be analysed.

The CONSORT Statement extensions for trials of non-pharmacological interventions and pragmatic intervention trials were used to design the study and will be used to report it.

### Sample size calculation

Sample size has been calculated to detect minimal significant effects on the variable of physical performance (SPPB): Accepting an alpha risk of 0.05 and a beta risk of 0.20 in a bilateral contrast, 318 individuals are required in order to detect a difference equal to or greater than 0.5 units in the SPPB[49, 50]. The common standard deviation has been taken to be 1.42. A drop-out rate of 20% is anticipated. Finally, 352 subjects have been included (n = 176 IG and n = 176 CG).

### Ethical aspects

Written informed consent has been obtained from all recruited subjects. Objectives, tests and other details about methodology and interventions were explained orally and in writing. The trial was approved by the Ethics Committee of the IDIAP Jordi Gol (code number P12/047) on June 1st, 2012. Funding from the Carlos III Health Institute was granted on December 20, 2012 (project code PI12/01503).

### Participants and recruitment

From February 2013 to January 2014, 370 individuals aged 65 years and over were recruited from 8 Primary Healthcare Centres (PHCC) located in two different districts of Barcelona. A total population of 33,857 aged 65 years and over live in the reference area.

Subjects were recruited by referral from the PHCC where the opportunity to participate in the study was offered on a regular daily basis to all patients meeting preliminary frailty criteria (Barber Questionnaire[51]). Eligibility was then verified with an assessment by a Case Management Nurse (CMN) through a personal interview. Participants meeting at least 3 Fried modified frailty criteria were included whilst those individuals with very slow or rapid gait speed, or cognitive impairment based on MEC-35 of Lobo[52], were excluded.

Inclusion and exclusion criteria are shown in Table 1.

**Figure 1:**
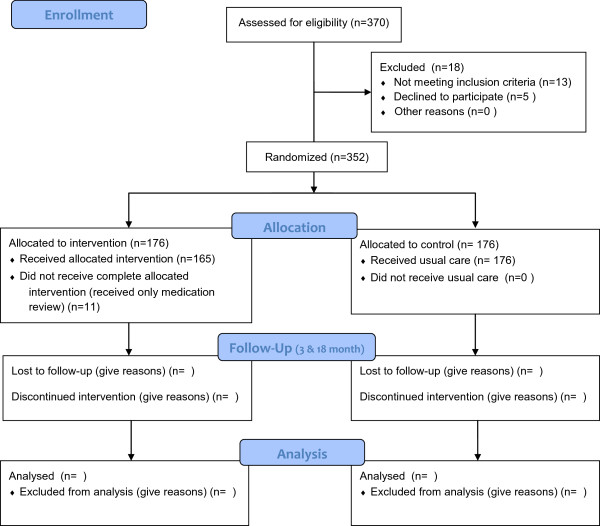
**Study flow diagram.**

The flow-chart of the trial according to CONSORT 2010 is visualized in Figure 1.

**Table 1:** **Inclusion and exclusion criteria**

Inclusion criteria	Exclusion criteria
• 65 years or older	• Medical conditions such as the presence of: unstable angina, uncontrolled congestive heart failure, unstable arrhythmia, COPD stage III or IV which contraindicate following a program of physical activity
• Resident in Barcelona, community-dwelling	
• Assigned to one of the 8 PHCC	
• Can attend on-site the consultation room at the PHCC	
• Will stay in the reference area a minimum of one year and a half	• Home Care Program or institutionalization at baseline. Planned admission to nursing home
• Frailty inclusion criteria:	• Participation in other physical activity program
• score of 1 point or above in the Barber Questionnaire	• Has been operated on hip and/or knee the last 6 month (walking independently with technical assistance is not a contraindication)
• Fried modified frailty criteria: 3 or more	
• Gait time between 10 to 30 seconds in the Timed Get Up and Go test	• Suffering a non-controlled neoplastic disease, terminal or severe disabling illness
• MEC-35 of Lobo ≥18 points (no severe cognitive impairment)	• Cannot understand Spanish
• Capable of consent. Agreement to participate in the study

Eligible patients who agreed to participate in the study were invited to sign the informed consent. Baseline variable collection was carried out by the CMN. Patients were then randomly assigned to the intervention and control groups. The computer-assisted simple randomization process was performed not by the recruiters but by an independent researcher. Random allocation sequence was implemented using sequentially numbered containers. Sequence was concealed until the interventions were assigned. Baseline and outcome measurements were blinded to group assignment. Follow-up evaluations are conducted by blind trained clinical researchers.

### Measures

Both cohorts receive identical baseline and follow-up evaluations. Table 2 shows the different time points when variables are measured.

**Table 2:** **Measurements at several time points**

	Selection	Baseline		After intervention		18 months	
	All	IG	CG	IG	CG	IG	CG
**FRAILTY MEASURES**							
Barber Questionnaire	x						
Fried modified criteria	x					x	x
SPPB		x	x	x	x	x	x
FRT		x	x	x	x	x	x
Unipodal Station		x	x	x	x	x	x
Strength of upper extremities		x	x	x	x	x	x
Strength of lower extremities		x	x	x	x	x	x
**FUNCTIONAL ASSESSMENT**							
Lawton & Brody Scale		x	x			x	x
Barthel Index		x	x			x	x
**NUTRITION**							
MNA		x	x			x	x
**COGNITIVE EVALUATION**							
MEC-35 of Lobo	x						
Short and Medium-Term Verbal Memory		x	x	x	x	x	x
Animal Naming Test		x	x	x	x	x	x
Evocation of words		x	x	x	x	x	x
Designation of famous people names		x	x	x	x	x	x
Verbal designation of images		x	x	x	x	x	x
Verbal abstraction of word pairs		x	x	x	x	x	x
**DRUGS and PRESCRIPTION**							
Total number of drugs		x	x	x	x	x	x
Psychotropic Medication presence		x	x	x	x	x	x
Withdrawal of drugs				x	x	x	x
**OTHER VARIABLES**							
Comorbidities		x	x			x	x
Biological measurements		x	x			x	x
Analytical parameters		x	x			x	x
Sphincter incontinence		x	x			x	x
Visual impairment		x	x			x	x
Auditive impairment		x	x			x	x
Technical support aids		x	x			x	x
Quality of Life: SF12		x	x			x	x
**ADVERSE OUTCOMES**							
Falls		x	x			x	x
Fractures		x	x			x	x
Hospital admissions		x	x			x	x
Home care inclusions						x	x
Institutionalizations						x	x
Death						x	x

1. Fried modified criteria for frailty [3] (three or more of the following criteria have to be present):
  Unintentional weight loss (3 kg in past 6 months)
  Self-reported exhaustion (2 questions from CES-D scale)
  Weakness (5 chair stand-up test, unable)
  Slow walking speed (more than 10 seconds) evaluated by Timed -up-and-Go test (TGUGT). This is a reliable test for quantifying functional mobility (lower extremities function) and for measuring balance (fall risk). The person may wear their usual footwear and can use any assistive device normally employed. The TGUGT is conducted using a chair with arms, and a seat height of 46 cm, placed upon a flat surface with a line marking the 3m turning point. Subjects are instructed on the word ‘go’, to get up and walk as quickly and as safely as possible to cross the line marked on the path, turn around, walk back to the chair and sit down again. The activity will be timed from the subject’s back leaving the back of the chair to the return of the subject to this same position.
  Low physical activity measured by the IPAQ Questionnaire[53].
2. Physical performance:
  2.1 *Short Physical Performance Battery*.
    The short physical performance battery (SPPB) is a simple standardised objective assessment tool of lower limb function[16] that tests standing balance, ability to repeatedly stand from a sitting position, and habitual gait speed. Each component is scored between 0–4 (total score 0–12) with higher scores indicating better functioning. In community-dwelling older adults, lower SPPB scores predict greater risk of mortality, nursing home admission, hospitalization, and incidence of disability. The SPPB consists of:
  2.1.1) *Balance test*
    Participants are asked to hold three increasingly challenging standing positions for 10 seconds each: (1) a side-by-side position, (2) semi-tandem position (the heel of one foot beside the big toe of the other foot), (3) tandem position (the heel of one foot in front of and touching the toes of the other foot).
  2.1.2) *Repeated chair stands test*
    This is performed using a straight-backed chair, placed with its back against a wall. Participants are first asked to stand from a sitting position without using their arms. If they are able to perform the task, they are then asked to stand up and sit down five times, as quickly as possible, with arms folded across their chest. The time to complete five stands is recorded and used for future analyses.
  2.1.3) *Gait speed* (*8 meters walk*)2.1.3)
  2.2 *Functional Reach Test* (FRT).
    This is a valuable test used to measure standing balance and stretching, detecting balance impairment over time. It can predict the risk of falling. After the examiner explains and shows the FRT, each subject performs 2 trial tests. Functional reach is measured by using a levelled yardstick attached to the wall at the height of the subject’s right acromion. To measure the subject’s reaching distance, an examiner stands 0,5 m away from the measuring tape and records the end reach position, checking that the initial position is correct: subjects stand comfortably with feet approximately shoulder-width apart, just before a line marked on the floor at the same level as the measuring tape beginning (0cm); participants then extend the right arm parallel to the yardstick and, without touching the wall, place the third metacarpal along the measuring tape and have to reach as far forward as they can without losing their balance (end position). Subjects are allowed to balance on their toes; however, touching the wall, stepping while reaching forward, or holding onto their clothing with the left hand invalidate the trial. If invalidated, the trial is repeated with a maximum of 2 tests more to achieve 2 valid trials. All subjects are protected during the test. The best result from the two attempts is recorded.
  2.3 *Unipodal station*
    The patient is placed in a standing position, arms crossed over the chest, with one leg used for support in an extended position, and the other slightly bent at the knee (there can be no contact between the two legs). Once placed in the correct position (eyes open), the chronometer is activated and then stopped when either the patient moves the foot used as a base or when 30 seconds have passed; two attempts with the same foot are made and the best result recorded.
3. Muscle strength.
  3.1 *Evaluation of upper extremities strength* is assessed through the measurement of force with a handgrip dynamometer. *Grip strength* is assessed using a portable hand dynamometer (JAMAR®0-90 kg, coding 506320). The participants are seated with their shoulder in a neutral position and their elbow flexed at 90°. Three attempts are performed alternately in each hand; the mean of the three measures is recorded.
  3.2 *Evaluation of lower extremities strength* is assessed through the bilateral measurement of the quadriceps muscle force using a digital dynamometer (Chronojump 1.4.5-1.4.6 Boscosystem® Encoder).
4. Activities of Daily Living (ADL) assessment:
  4.1 * Lawton & Brody Instrumental Activities of Daily Living scale*[54].
    This is an instrument assessing independent living skills which are considered more complex than the basic activities of daily living. The instrument is most useful for identifying how a person is functioning at the present and for observing improvement or deterioration over time. There are 8 domains of function measured with the Lawton & Brody scale. Women have been traditionally assessed in all 8 areas of function whilst men have not been asked about the domains of food preparation, housekeeping, and laundering. Individuals are scored according to their highest level of functioning in that category. A summary score ranges from 0 (low function, dependent) to 8 (high function, independent).
  4.2 *Barthel Index of Basic Activities of Daily Living*[55].
    First developed in 1965, it measures functional disability by quantifying patient performance in 10 activities of daily life. These activities can be grouped according to self-care (feeding, grooming, bathing, dressing, bowel and bladder care, and toilet use) and mobility (ambulation, transfers, and stair climbing). 5-point increments are used in scoring, with a maximal score of 100 indicating that a patient is fully independent in physical functioning, and a lowest score of 0 representing a totally dependent bed-ridden state.
5. Nutritional Assessment:
  *Mini Nutritional Assessment* MNA®[56]. The MNA consists of four parts: anthropometric measurements, general status, diet information, and subjective assessment. A score of less than 17 points (out of a maximum of 30) is regarded as an indication of malnutrition, 17–23.5 points indicate a risk of malnutrition and >23.5 points indicate that the person is well nourished.
6. Neuropsychologist Performance:
  The first neuro-psychometric instrument developed in Spain to measure semi-quantitatively cognitive status in clinical neurology was the Barcelona Test (BT). A shortened version of the BT, named Barcelona Test Review[57], is used for neuropsychological area evaluation in our participants and it takes only 20–30 minutes to administer. Tests applied are:
  6.1. *Short and Medium*-*Term Verbal Memory* is the capacity to hold a small amount of information in the mind in an active, readily available state for a short period of time (seconds) and medium-term period of time (minutes). For the condition referred to as short term, subjects will be instructed to listen to a little story text, 21-pieced-sentences (elements), read by a blind evaluator; once finished, the patient will be asked to repeat the general content and as many details as he or she can remember; the duration of short-term memory is believed to be in the order of seconds. Then, the participant will be submitted to other cognitive trials to divert attention. After 25 minutes, the subject is directly asked for the story again to evaluate medium-term verbal memory. A commonly cited capacity of short and medium-term is 7±2 elements.
  6.2. * Animal Naming Test* consists of asking the patient to name as many animals as possible in one minute. A blind evaluator must write down the answers, so they can be checked for duplicate responses (repeated words invalidate one of them). The goal of this test is to score at least 14.
  6.3. * Evocation of words* beginning with one explicit letter, similar to the previous test, in this case the participant will tell the examiner as many words beginning with “p” as possible in three minutes (repeated words invalidate one of them). All kinds of words are allowed, except plurals or the masculine and feminine of the same word, conjugating verbs, and diminutives. The goal of this test is to score at least 27.
  6.4. *   Designation of famous people names*, the identification of 30 famous faces and their corresponding name permits an examination of the semantic brain area and can identify a possible clinical syndrome of prosopagnosia. Evaluated by “success”, “failure” or “tip of the tongue (TOT) phenomenon”, goal is 23 success, 3 failure, 4 TOT.
  6.5. *   Verbal designation of images*. Fourteen pictures of different objects or animals are presented to participants and they have to identify each name as quickly as they can: if subjects guess the name between 0–3 seconds this signifies 3 points, between 3–10 seconds, 2 points, and if takes 10–30 seconds it represents 1 point. If the patient does not recognize the picture-name, it is equivalent to 0 points. Goal of the test is to score 41 points.
  6.6. *  Verbal abstraction of word pairs*, also called “Similarities – Abstraction”, explores patients’ concept formation ability, as the participant must “extract” the common abstract element that links the two words featured. Through this test the ability to discriminate “concrete thinking “from” abstract thought” can be evaluated. The goal of this test is to score at least 5 of 6 pair of words.
7. Medication
  7.1. Number of prescribed drugs.
  7.2. Number of prescribed benzodiazepines.
  7.3. Presence of antidepressants (yes/no).
  7.4. Withdrawal of drugs (yes/no).
  7.5. Number of drugs retired at the closing date of the study.
8. Quality of life. 12-Item Short-Form Health Survey (SF-12) [58].
9. Adverse Outcomes: Falls, fractures, hospital admissions, institutionalization, inclusion in a Home-Care Program, or death.

### Independent variables

Age. Gender. Marital status. Cohabitation. Education Level. Socioeconomic status. Existence of elevator in the building. Provision of regular company.

Co-morbidities assessed in the clinical record: osteoarthritis, fractures in the last 5 years (hip fracture specified), presence of prosthetic joints, vision impairment, hearing impairment, cardiovascular diseases (hypertension, stroke, ischemic heart disease, arrhythmia, congestive heart failure, intermittent claudication, chronic venous insufficiency), pulmonary diseases (Chronic Obstructive Pulmonary Disease (COPD), asthma), endocrinology diseases (diabetes, dyslipidemia, obesity, hypothyroidism, hyperthyroidsim), hematological (anemia), neurologic (Parkinson’s disease), psychiatric (anxiety, depression), chronic kidney disease.

- Comorbidity measured with Charlson Index [59].
- Biological variables: weight, height, body mass index, waist circumference, blood pressure.
- Analytical variables: hemoglobin, serum lipid profile, serum protein, serum albumin, glomerular filtration rate, plasma creatinine, glycated hemoglobin, ferritin, iron, vitamin D, vitamin B12, folic acid.
- Incontinence (urinary, fecal, both).
- Urinary catheter (yes/no).
- Wearing a diaper (yes/no).
- Usual sensation of light-headedness.
- Smoking (non-smoker, ex-smoker, current smoker).
- Devices for mobility (cane, walker).
- Falls, fractures, and hospitalizations in the previous year.

#### Intervention

The intervention consists of a triple disability preventive therapy, consecutively applied to each subject in the intervention group, in groups of 16 participants (see Table 3):

**Table 3:** **Description of Interventions**

3.1 Description of the rehabilitation therapy			
Basic exercise	Alternative exercise		Muscle group
▪ Chest Press against elastic resistance - sitting on a chair	▪ Chest Press against the wall		▪ Pectoral muscles
▪ Reverse Butterfly against elastic resistance - sitting on a chair			▪ Upper back muscles
▪ Arm press against elastic resistance - sitting on a chair	▪ Arm press against elastic resistance - standing position		▪ Muscles of the arms, and shoulders
▪ Stand up with palms on thighs - sitting on a chair	▪ Stand up using hand weighs - sitting on a chair		▪ Quadricep, hamstring, and gluteal muscles
▪ Lift the legs with hands on hips - sitting on a chair			▪ Hip flexor muscles
▪ Hip abduction/adduction - sitting on a chair	▪ Hip abduction/adduction - standing position		▪ Abductor/Adductor muscles
▪ Knee flexion - sitting on a chair	▪ Knee flexion - standing position		▪ Hamstring muscles
▪ Knee extension - sitting on a chair			▪Quadricep muscles
▪ Heel raises - sitting on a chair	▪ Heel raises - standing position		▪ Gastrocnemius and soleus muscles
**3.2 Description of the memory workshops**			
**Memory**	**Language**	**Sensory activation**	**Reasoning and calculation**
Short and Long-Term Visual Memory	Evocation of words beginning with different letters	Series of logical visual pattern recognition	Gnosia and praxia different developing techniques (reproduction of pragmatic models)
Short and Long-Term Written Memory	Crosswords	Marking edge of silhouettes	Letters and numbers matching through Maze Paths
Short and Long-Term Oral Memory	Completeness of unfinished sentences	Coloring components of Hidden Figures Test	Executive functions enhancing: abstract concepts of similar but different objects
Short and Long-Term Musical Memory	Oral communication with clue words	Spot the differences between two pictures	Identification of the inappropriate word in a pool of words
Working memory: identification of hidden figures test	Word search	Picture copies execution	Reading and exclusion of senseless sentences
Memorize an image and draw it from memory	Synonyms and antonyms	Objects, materials and sounds recognition with closed eyes	Filling the gap
True/False sentences	Matching words and their meaning	Group interaction by singing and musical performances	Numerical skills practice: operations and mental agility
Logos recognition	Visual-verb generation task: denomination of images, objects, parts of the human body	Famous faces recognition	
Geographical memory practice	Rearrange letters to form a word and rearrange words to form grammatical sentences		
**3.3 Description of the polymedication review**			
**Who does the intervention?**	**What are the objectives and criteria?**		**How is the intervention performed?**
2 doctors from the Project Group.	To reduce drug prescription of polymedicated patients* if possible, following :		A personalized e-mail is sent to each GP responsible for the patient participating in the intervention group throughout the first week of patient inclusion.
	-Stopp criteria,		
	Depending on the baseline drug prescription at the beginning of the study.		Every e-mail considers the individual profile of the patient referred and tries to adapt the general criteria to each particular case.
			The GP who regularly attends the intervention patient performs both reduction and re-education of unnecessary drugs. This approach should be done in a maximum of 3 clinical interviews specifically designed for this subject.
			E-mail content suggests the most recommended changes but the final decision corresponds to the discretion of the physician responsible for the patient.

*Polymedicated Patient: one that takes more than five drugs daily and continuously for a period not less than six months.

1. Rehabilitation therapy plus the posterior intake of 1 hyperproteic nutritional shake which is then taken daily for 1 month. All patients in the intervention group perform the aerobics exercise plan in the primary care centre, 60-minute session twice a week on non-consecutive days for 6 weeks (12 sessions of 60 minutes each). Subjects must incorporate a progressive increase in the intensity of the exercise in each session. One session a week is dedicated to work with balance and the other to strength training. Both balance and strength are based on functional exercises. All sessions begin with a warm up for 5 minutes, and end by cooling off for another 5 minutes with relaxing exercises. The sessions are conducted under the supervision of a specialist in physical activity. A hyperproteic nutritional shake is provided at the end of each session, and the amount of shakes needed for one month’s consumption post-physical therapy is assigned. The safety of the exercise program is measured by reviewing the record sheet for each patient in the training program, ascertaining cardiovascular decompensation and musculoskeletal injuries.
2. Memory workshops. Two speech therapists from the rehabilitation unit conduct 12 sessions of practical exercises (written, oral, corporal, and musical) in groups of 16 participants. Each of the 12 sessions lasts 90 minutes and is conducted twice a week. Each person in the intervention group has their own material to work short and long-term memory, with exercises for the identification of figures and images, evocation of words, true or false sentences, crosswords, completion of unfinished sentences, and other language exercises such as synonyms and antonyms.
3. Medication Review. Reduction of potentially inappropriate medications, especially in polymedicated patients, after review from general practitioners. A patient is considered to be polymedicated when taking more than five medications daily and continuously for a period not less than six months. Medication review follows the Screening Tool of Older Persons’ potentially inappropriate Prescriptions (STOPP) criteria [60]. In addition to the review of medication, verbal guidance on each of the drugs consumed is also provided. After an e-mail sent with the changes suggested by two doctors from the Project Group, this intervention is carried out by every patient’s general practitioner, during the first month of the intervention, in a maximum of 3 clinical interviews for that purpose. It especially focuses on reducing the consumption of benzodiazepines or other psychotropic drugs.

The intervention group also receives two group sessions regarding dietary advice, lifestyles, and home hazards.

#### Control group

Subjects in the control group continue with their daily activities and receive regular monitoring and treatment of their diseases by their general practitioners. They are also invited to two group sessions regarding dietary advice, lifestyles, and home hazards.

### Statistics

Intention to treat analyses will be performed. Baseline characteristics will be compared between groups by independent t tests and Chi-square tests. Outcome variables will be calculated for each individual and time point (difference between the result of SPPB, muscle strength, and other frailty variables in each time point and the initial value), and 95% confidence intervals for the differences between groups will be calculated. Data will be analysed using repeated measures analysis of variance (ANOVA) consisting of intervention and control groups and time (baseline, post-intervention, follow-up).

Also, for longitudinal adverse outcome measures (disability, home care inclusion, institutionalization or death), survival analyses using Cox’s regression models will be applied. The statistical significance level will be set at p <0.05.

## Discussion

Our study is addressed at evaluating the effectiveness of a multifactorial intervention to improve frailty parameters and prevent disability in patients 65 years or older. Improvements in physical performance, muscle strength, nutritional status, and cognitive performance are expected, as well as a reduction in the incidence of new complications such as falls, fractures, hospital admissions, and worsening of ADL scales, all of which are related to the appearance of disability[33, 34]. Tackling frailty in a multifaceted manner will also diminish adverse outcomes such as inclusion in a home-care program, institutionalization or death.

In the field of preventive geriatrics, studies have shown that exercise training has clinical benefits inducing positive physiologic changes in muscle and function while multi-nutrient supplementation alone, without concomitant exercise, does not reduce muscle weakness or physical frailty[61].

The innovation of our study lies in regard to the follow-up and evaluation of a multifaceted strategy focused on different risk factors: physical decline, cognitive impairment, nutritional status, and polypharmacy. Previous series have provided a certain degree of evidence about improvement with these interventions on only an individual basis.

The greatest limitation of this study could proceed from the lack of agreement in the scientific community with respect to the definition of frailty and the most suitable measurements to gauge it. Including non- frail subjects (non-homogeneous risk state) could affect generalizability. The initial inclusion criterion, the low specificity Barber Questionnaire, has been included in this study because it was the first frailty test to be used in our clinical records. It has been also complemented, however, with other inclusion criteria such as the TGUGT. Participants scoring lower than 10 seconds or higher than 30 are excluded as they are considered either too frail or not frail enough to benefit from the intervention. The exclusion of more severely affected frail patients, because of their poor physical or cognitive condition, may limit external validity. Nevertheless, the random distribution of our patients to both groups guarantees comparability. Also, additional information about potential confounders (comorbidity, sensory impairment, and social risk) and the use of other parameters and tests of frailty are expected to solve the possible selection bias and help to further characterize the study population. Losses to follow-up are minimized through contacting the participants by telephone.

If evidence of a multi-strategy composed of physical exercise and a cognitive workshop, along with nutritional support and medication review, is achieved as an effective approach, a future implementation should be considered as a Frail-Community Prevention Program for the elderly to prevent or delay disability.

## Abbreviations

MEC-35: Cognition Mini-Exam of Lobo
SD: Standard deviation
CES-D: Center for Epidemiologic Studies Depression Scale
SPPB: Short Physical Performance Battery
IG: Intervention group
CG: Control group
CONSORT: Consolidated standards of reporting trials
PHCC: Primary Health Care Centre
CMN: Case Management Nurse
TGUGT: Timed get up and go test
FRT: Functional reach test
ADL: Activities of daily living
MNA: Mini Nutritional Assessment
BT: Barcelona Test
SF-12: 12-Item Short-Form Health Survey
COPD: Chronic obstructive pulmonary disease
STOPP: Screening Tool of Older Persons’ potentially inappropriate Prescriptions.
